# Mechanistic insights into the inhibitory effects of eukaryotically expressed Sarcotoxin II 50 on *Klebsiella pneumoniae* invasion into pulmonary epithelial cells

**DOI:** 10.1128/spectrum.00962-25

**Published:** 2026-03-17

**Authors:** Wen-Xia Liu, Gong-You Zhang, Hong-Hai Ke, Xue-Ting Zhang, Yu-Ling Shi, Meng-Zhu Liu, Su-Wen Yang, Yong-Xin Yang, Ying-Qian Kang, Hong-Mei Liu, Bing Wang

**Affiliations:** 1 Engineering Research Center of Health Medicine biotechnology of Institution of higher education of Guizhou Province / Engineering Research Center of Medical Biotechnology, Guizhou Medical University, School of Biology and Engineering (Modern Industry College of Health Medicine) / Basic Medical College 74628, Guiyang, Guizhou, China; 2 Key Laboratory of Environmental Pollution Monitoring and Disease Control, School of Public Health, China Ministry of Education (Guizhou Medical University), Guiyang, Guizhou, China; 3 People’s Hospital of Kai yang County, Guiyang, Guizhou, China; 4 Laboratory Animal Center of Guizhou Medical University, Guiyang, Guizhou, China; 5 Guizhou University of Traditional Chinese Medicine, Guiyang, Guizhou, China.; Icahn School of Medicine at Mount Sinai, New York, New York, USA

**Keywords:** AMP, hvKP, cKP, antimicrobial activity, Sarcotoxin II 50

## Abstract

Hypervirulent (*hvKP*) and classical (*cKP*) strains of *Klebsiella pneumoniae* are major contributors to serious hospital-acquired infections, and the growing issue of antibiotic resistance highlights the urgent need for new antimicrobial approaches. In this study, we investigated the inhibitory effects of Sarcotoxin II 50, an insect-derived antimicrobial peptide, on *K. pneumoniae*. Our findings show that Sarcotoxin II 50 exerts potent antibacterial activity by impairing capsular polysaccharide (CPS) production and fimbrial formation, which compromises bacterial adhesion and colonization capacity. *In vitro* experiments demonstrated that Sarcotoxin II 50 effectively suppressed the growth of *cKP*. Mechanistic analyses revealed that the peptide interferes with CPS biosynthesis and alters the structure and expression of type 1 fimbriae (*fimA*) and type 3 fimbriae (*mrkA*, *mrkD*, *mrkH*), which are associated with reduced bacterial tissue invasion and attenuated virulence in a murine abscess model. Overall, this study provides a detailed mechanistic understanding of how Sarcotoxin II 50 inhibits *K. pneumoniae* both *in vitro* and *in vivo* under the tested experimental conditions. These results offer a mechanistic basis for the development of novel antimicrobial therapies targeting *K. pneumoniae* infections and support the further exploration of this peptide as a promising antimicrobial candidate. Characterization of a new insect peptide, Sarcotoxin II 50, with anti-*K*. *pneumoniae* activity from the housefly for the first time.

**IMPORTANCE:**

Sarcotoxin II 50 inhibited *K. pneumoniae* invasion into A549 and BEAS-2B lung epithelial cells. Sarcotoxin II 50 inhibits *K. pneumoniae* invasion into epithelial cells by affecting capsular polysaccharide synthesis, fimbriae formation, and cell division. This manuscript investigates, for the first time, the function and mechanism of the Sacrotoxin II family member in combating *K. pneumoniae*, 40 years after its initial identification.

## INTRODUCTION

*K. pneumoniae* is a leading cause of hospital-acquired pneumonia (1), posing a serious challenge to healthcare systems worldwide due to its high pathogenicity and associated mortality (2). This bacterium commonly causes pneumonia, urinary tract infections, and sepsis, particularly in immunocompromised patients (3, 4). In contrast, hypervirulent *K. pneumoniae* (hvKP) is more often associated with community-acquired infections and has the ability to disseminate to multiple organs and tissues (5). Due to its heightened virulence and rising antimicrobial resistance, hvKP is linked to severe invasive diseases, such as sepsis, meningitis, and liver abscesses (6–8), drawing increasing attention in recent years (4). Its strong virulence and high transmissibility have made hvKP a major global public health concern (9, 10). Compared with classical *K. pneumoniae* (cKP), hvKP shows markedly enhanced invasiveness and resistance, largely driven by its hypermucoviscous phenotype, increased production of capsular polysaccharides, and virulence plasmid–mediated immune evasion (11, 12). Although current antibiotics can partially control *K. pneumoniae* infections, strategies that specifically target hvKP virulence determinants remain limited, creating significant challenges for clinical management (13). In particular, the hvKP strain CG43 has attracted attention due to the high prevalence of genes involved in capsular polysaccharide biosynthesis in clinical isolates (14, 15). Therefore, the development of new antimicrobial agents that can modulate bacterial virulence represents a critical avenue for addressing the growing threat of antimicrobial-resistant *K. pneumoniae*.

Capsular polysaccharide (CPS) is a key virulence factor that plays a central role in *K. pneumoniae* pathogenicity (16–18). It contributes to bacterial survival and disease progression by mediating immune evasion through multiple mechanisms (19). CPS forms a protective layer around the bacterium that inhibits phagocytosis by host immune cells and reduces the efficacy of antimicrobial agents, with the level of protection closely linked to capsule thickness (20). In hypervirulent *K. pneumoniae* (hvKP), the *rmpA2* gene, located on large virulence plasmids, encodes a transcriptional activator belonging to the mucoid phenotype regulatory family. *rmpA2* enhances the expression of CPS biosynthetic gene clusters, leading to excessive capsule production and the development of a thick, mucoid layer on the bacterial surface (21). This overproduction of CPS underlies the characteristic hypermucoviscous phenotype, which significantly increases bacterial adhesion and colonization within host tissues while providing strong resistance to complement-mediated killing and phagocytosis. Studies in murine models of pneumonia, intraperitoneal infection, and urinary tract infection have confirmed a direct link between *rmpA2*-mediated capsule overproduction and elevated bacterial virulence. hvKP strains regulated by *rmpA2* produce abundant CPS (22), resulting in a hypermucoviscous phenotype (23, 24), enhanced tissue colonization, and increased resistance to immune clearance (25–28). These findings have been consistently validated across multiple infection models (19, 29). Beyond CPS, fimbrial-associated genes also contribute to cell envelope biogenesis and together help establish antimicrobial tolerance barriers (30), often regulated through quorum-sensing mechanisms. The *wzc* gene encodes a key regulator of capsular protein synthesis and is essential for maintaining the hypermucoviscous phenotype; deletion of *wzc* reduces mucoidy, impairs biofilm formation, and diminishes bacterial adherence to host cells (31). Similarly, proteins encoded by *galF* and *wzc* are involved in CPS translocation and proper capsule assembly on the bacterial surface. Given the feasibility of targeting CPS for therapeutic purposes (26), understanding these structure–function relationships provides important insights into bacterial resistance mechanisms and offers a strong theoretical foundation for developing novel antimicrobial strategies and advancing our knowledge of hvKP pathogenesis.

Insects are a rich source of novel antimicrobial peptides (AMPs) (32), having evolved sophisticated humoral and cellular defense systems that efficiently protect them against a wide range of pathogens (33). These peptides are generally cationic, enabling them to interact specifically with negatively charged components on microbial surfaces, thereby exerting antimicrobial effects (34–36). As potential alternatives to conventional antibiotics, AMPs have garnered considerable attention due to their broad-spectrum activity and relatively low propensity to induce resistance (37), making them a key focus in the search for new strategies to prevent and treat bacterial infections (38, 39). Sarcotoxin IIA (STX IIA) is an inducible antimicrobial protein derived from the hemolymph of *Sarcophaga peregrina* larvae and belongs to the Sarcotoxin II family. Its cDNA was first cloned and characterized by Ando and Natori, who showed that STX IIA is highly expressed in response to infection or tissue injury and plays a central role in the insect’s humoral immune defense (40). Genes encoding these peptides often occur in tandem arrays and are co-activated during immune challenges, with predominant expression in the insect fat body, underscoring their importance in innate immunity (41). Functionally, Sarcotoxin IIA has been reported to inhibit actively growing Gram-negative bacteria. At a concentration of 25 µg/mL, it can disrupt cell wall synthesis in *Escherichia coli* and induce distinctive morphological changes, such as cell elongation and surface protrusions, suggesting that its primary mechanism involves interference with bacterial cell wall or septum formation (42). Structurally, Sarcotoxin IIA consists of approximately 270 amino acids, and its molecular weight and conformation differ significantly from short-chain cecropin-type AMPs. Classical purification studies have confirmed that members of the Sarcotoxin II family, including the IIA variant, can be isolated as active antimicrobial components from the hemolymph of immunized larvae. These observations indicate that Sarcotoxin IIA expression is tightly regulated by immune stimuli and is rapidly upregulated in the fat body following pathogen infection, contributing to the insect’s innate immune defense (43). Given that many pathogenic bacteria depend on intact capsules or cell walls to maintain virulence, Sarcotoxin IIA and its engineered derivatives—such as Sarcotoxin II 50 investigated in this study—may represent promising candidates for targeting bacterial virulence factors and offer valuable insights for developing novel anti-infective therapies.

In this study, Sarcotoxin II 50 was isolated from houseflies subjected to body wall injury. We demonstrated that eukaryotic expression of Sarcotoxin II 50 inhibited the growth of *K. pneumoniae*. Moreover, Sarcotoxin II 50 markedly reduced the ability of *K. pneumoniae* to invade A549 and BEAS-2B cells and decreased the expression of capsular polysaccharide and other key virulence genes. *In vivo* experiments further showed that hvKP strains treated with Sarcotoxin II 50 induced significantly smaller abscesses in mice, indicating its inhibitory effect against hypervirulent strains. Together, these results highlight Sarcotoxin II 50 as a promising candidate for the treatment of infections caused by both hvKP and cKP and provide a solid theoretical basis for future research on antimicrobial peptide-based therapies.

## MATERIALS AND METHODS

### Bacterial strains and culture conditions

*K. pneumoniae* ATCC 27736, a panel of clinical *K. pneumoniae* isolates (ATCC 21005, 25003, 25017, 26001, 25005, 25010, 26010, 26020, and 26039), and *Escherichia coli* ATCC 25922 were cultured overnight in Luria–Bertani (LB) broth at 37°C with shaking at 200 rpm. For subsequent experiments, bacterial cultures were plated onto tryptic soy agar (TSA) or grown in tryptic soy broth (TSB) as required.

### cDNA cloning and sequence, structural modeling of Sarcotoxin II 50

Sarcotoxin II 50 was amplified with PCR from *E. coli*-stimulated housefly 3rd larval cDNA pool. Its sequence had been submitted to NCBI GenBank with GenBank BankIt Submission ID: 2,936,343 (GenBank accession number BankIt2936343 Musca PV296360). The open reading frame (ORF) was analyzed by DNAMAN ORF Finder. The full-length ORF of Sarcotoxin II 50 was inserted into PcDNA3.1(+) vector with the BamH I and NotI cleavage sites for eukaryotic expression. The signal peptide was analyzed by SignalP 6.0 online software (SignalP - 6.0 - Services - DTU Health Tech). Homologs of Sarcotoxin II 50 and their sequence alignment were performed using BioEdit software. The structure of Sarcotoxin II 50 was minimized using AlphaFold2 online software.

### Cell culture

Human lung adenocarcinoma A549 cells were kindly provided by the School of Public Health, Guizhou Medical University, and normal human bronchial epithelial BEAS-2B cells were obtained from the School of Modern Industry and Health Medicine at our institution. Both A549 and BEAS-2B cells were cultured in 25 cm² flasks containing Dulbecco’s Modified Eagle’s Medium (DMEM; Gibco) supplemented with 10% fetal bovine serum (FBS; Gibco) and 100 μg/mL penicillin–streptomycin, and incubated at 37°C in a humidified atmosphere with 5% CO₂. When cells reached approximately 80% confluence, they were detached using 0.25% trypsin–0.53 mM EDTA (Gibco) and seeded into six-well plates at 5 × 10⁵ cells per well or 24-well plates at 8 × 10⁴ cells per well for transfection and subsequent experiments.

### Cell transfection procedure

Cells were cultured to approximately 80% confluence prior to transfection. Transfections were carried out using Lipofectamine 2000 in accordance with the manufacturer’s instructions. A549 and BEAS-2B cells were divided into two groups: one transfected with the Sarcotoxin II 50 expression plasmid and the other with the pcDNA3.1(+) vector as a control. Before transfection, the culture medium was replaced with Opti-MEM. For six-well plates, 4 μg of plasmid DNA was diluted in 200 μL of Opti-MEM and mixed gently. Separately, 8 μL of Lipofectamine 2000 was diluted in 200 μL of Opti-MEM and incubated at room temperature for 5 min. The DNA and Lipofectamine solutions were then combined and incubated for 20 min at room temperature to allow complex formation, after which the complexes were added dropwise to the cells with gentle mixing. For 24-well plates, the procedure was scaled proportionally using 1 μg of plasmid DNA and 2 μL of Lipofectamine 2000 per well. For transfection in 6-well plates, plasmid DNA (4 µg/well+8µL Lipofectamine 2000 / well) ; 96-well plates, plasmid DNA (0.2 µg) was combined with 0.8 µL of a liposome-based transfection reagent. Following incubation at 37°C in a 5% CO₂ atmosphere for 6 h, the transfection medium was replaced with DMEM containing 10% FBS and no antibiotics, and the cells were further cultured for downstream experiments.

### CCK-8 cell viability assay

To determine whether Sarcotoxin II 50 transfection affects host cell viability, the Cell Counting Kit-8 (CCK-8) assay was performed. Cells were seeded in 96-well plates at a density of 5 × 10³ cells per well and incubated overnight to allow adherence. Cells were then transfected with either the Sarcotoxin II 50 expression plasmid (S50) or the empty pcDNA3.1 vector, following the transfection procedure described above. At 24, 29, 36, and 48 h post-transfection, 10 μL of CCK-8 reagent was added to each well containing 100 μL of culture medium, and the plates were incubated at 37°C for 1–2 h. Absorbance was subsequently measured at 450 nm using a microplate reader to assess cell viability.

### Inhibition of *K. pneumoniae* invasion by Sarcotoxin II 50

For the invasion assay, *K. pneumoniae* cells were first washed with sterile saline, and the multiplicity of infection (MOI) was adjusted to 20. Bacteria were then added to host cells and incubated at 37°C with 5% CO₂ for 5 h. Following incubation, the cells were washed with PBS and treated with gentamicin (40 μg/mL) to eliminate extracellular bacteria. Cells were washed twice with PBS, digested with 100 μL of 0.25% trypsin–0.53 mM EDTA, and lysed using 500 μL of 0.1% Triton X-100 for 10 min in a cell culture incubator. The released *K. pneumoniae* were collected by centrifugation and resuspended in 180 μL PBS. For CCK-8-based quantification, 20 μL of CCK-8 reagent was added to each well and incubated at 37°C in the dark for 1 h, followed by absorbance measurement at 450 nm using a microplate reader. Intracellular bacterial counts were also determined by plating the lysates on TSB agar and enumerating colony-forming units (CFU). All experiments were performed in triplicate.

### Expression analysis of Sarcotoxin II 50 in A549 and BEAS-2B cells

Sarcotoxin II 50 is a heterologous gene, and its presence in A549 and BEAS-2B cells represents exogenous expression rather than endogenous activity. To confirm successful transfection and expression, total RNA was extracted from A549 and BEAS-2B cells at 1.5, 6, 12, 24, and 36 h post-transfection, as well as at 5 h post-infection, using TRIzol reagent. DNase I treatment was applied to remove any residual plasmid DNA. Complementary DNA (cDNA) was synthesized using the PrimeScript RT reagent kit with gDNA Eraser (Wuhan Bogede Biotechnology Co., Ltd.), and Sarcotoxin II 50 expression levels were quantified by real-time quantitative PCR (RT-qPCR). GAPDH served as the internal reference, and the 1.5 h post-transfection group was used as the baseline control. The PCR cycling program was set as follows: 95°C for 30 s, followed by 40 cycles of 95°C for 10 s, 60°C for 30 s, and 72°C for 30 s. Relative gene expression was calculated using the 2^–ΔΔCt^ method (44, 45). Primer sequences are provided in Table S1.

### Sarcotoxin II 50 inhibits intracellular *Klebsiella pneumoniae* growth

A 6-hour growth curve assay was conducted to assess the impact of Sarcotoxin II 50 on the replication of intracellular *K. pneumoniae* within A549 and BEAS-2B cells. Transfected cells were infected with *K. pneumoniae* at a multiplicity of infection (MOI) of 20 and incubated for 5 h to allow bacterial internalization. After incubation, cells were washed with PBS and treated with gentamicin (40 μg/mL) to eliminate extracellular bacteria. The plates were then incubated at 37°C in 5% CO₂ for 20 min and washed twice with PBS. Cells were subsequently detached using trypsin and 0.53 mM EDTA and lysed with 0.1% Triton X-100 to release intracellular bacteria. The lysates were centrifuged at 3,000 rpm for 3 min, and the resulting bacterial pellets were resuspended in 200 μL of LB broth. Bacterial growth was monitored by measuring the optical density at 595 nm (OD₅₉₅) hourly. Growth curves were plotted with OD₅₉₅ on the y-axis against time to visualize intracellular bacterial proliferation.

### Sarcotoxin II 50 alleviates *hvKP 26020*-induced subcutaneous abscess infection in mice

Due to the challenges in quantitatively assessing bacterial colonization and abscess formation in lung tissue using conventional pulmonary infection models (46, 47), as well as the rapid disease progression and high variability of endpoints in these models (48), a mouse subcutaneous infection model was employed. This approach allows direct assessment of abscess size, bacterial burden, and related parameters, providing a reliable measure of the *in vivo* antibacterial activity of Sarcotoxin II 50. For the subcutaneous infection assay, intracellular *K. pneumoniae* were harvested from A549 or BEAS-2B cells transfected with either Sarcotoxin II 50 or the pcDNA3.1(+) vector. Bacterial suspensions from experimental and control groups were standardized using a microplate reader and adjusted to equivalent concentrations based on optical density measurements. Each mouse received a subcutaneous injection of 100 μL of the calibrated bacterial suspension. Clinical signs and abscess formation were monitored every 12 h. At 48 h post-infection, abscess tissues were aseptically collected, homogenized in 1 mL of sterile saline, serially diluted, and plated for enumeration of colony-forming units (CFU).

### ELISA assessment of Sarcotoxin II 50 on cellular inflammatory responses

Cells were seeded into six-well plates at a density of 4 × 10⁵ cells per well. At 24 h post-transfection, cells were infected with a *K. pneumoniae* suspension at a multiplicity of infection (MOI) of 20 and incubated for 5 h at 37°C. After incubation in a humidified 5% CO₂ atmosphere, culture supernatants and cells were collected and centrifuged at 500 × *g* for 5 min. Concentrations of interleukin-6 (IL-6) and tumor necrosis factor-alpha (TNF-α) in the supernatants were measured using ELISA kits according to the manufacturer’s protocols.

### Assessment of Sarcotoxin II 50 on *K. pneumoniae* capsular polysaccharide

The capsular polysaccharide (CPS) content of intracellular *K. pneumoniae* was determined using the resorcinol–sulfuric acid colorimetric assay (49). Before measurement, intracellular bacterial counts in the control (pcDNA3.1(+)) and Sarcotoxin II 50 groups were normalized using a microplate reader to ensure equal bacterial numbers. Bacterial suspensions were centrifuged, resuspended in 100 μL of DEPC-treated water, and kept on ice. Subsequently, 500 μL of concentrated boric acid–sulfuric acid solution was added, and the mixture was boiled in a water bath for 5 min. After cooling slightly, 5 μL of 0.15% resorcinol–0.5% NaOH solution was added and thoroughly mixed. Absorbance was measured at 525 nm. For the standard curve, 10 Eppendorf tubes containing different concentrations of glucuronic acid were prepared, with DEPC-treated water added to a final volume of 100 μL. These samples underwent the same procedure. The CPS content of intracellular bacteria was then calculated by interpolating the measured absorbance values against the standard curve. All assays were performed in triplicate.

### Scanning electron microscopy (SEM)

Intracellular *K. pneumoniae* were collected as described above and harvested by centrifugation at 3,000 rpm for 3 min. The bacterial pellets were then fixed in 2.5% glutaraldehyde at 4°C for 24 h. SEM imaging was subsequently performed following established protocols (44).

### Total RNA extraction and real-time PCR analysis of *K. pneumoniae* gene expression

Transfected cells were infected with *K. pneumoniae* at a multiplicity of infection (MOI) of 20 and incubated at 37°C for 5 h. To isolate intracellular bacteria, the culture medium was discarded, and cells were washed twice with PBS. Extracellular bacteria adhering to the monolayer were eliminated by treatment with gentamicin (40 μg/mL) for 20 min at 37°C in a 5% CO₂ atmosphere, followed by two additional PBS washes. The cell monolayer was then digested with 0.25% trypsin–0.53 mM EDTA and lysed with 0.1% Triton X-100 for 10 min in a cell culture incubator. Intracellular bacteria were collected by centrifugation at 12,000 rpm for 5 min. Total RNA was extracted using TRIzol reagent and reverse-transcribed into cDNA with the PrimeScript RT Reagent Kit containing gDNA Eraser. Gene expression was quantified by real-time PCR using 16S rRNA as an internal reference to normalize for host cell contributions. PCR cycling conditions and statistical analyses were performed as described above. Primer sequences are provided in Table S2.

## RESULT

### Characterization of housefly Sarcotoxin II 50

**Fig 1:**
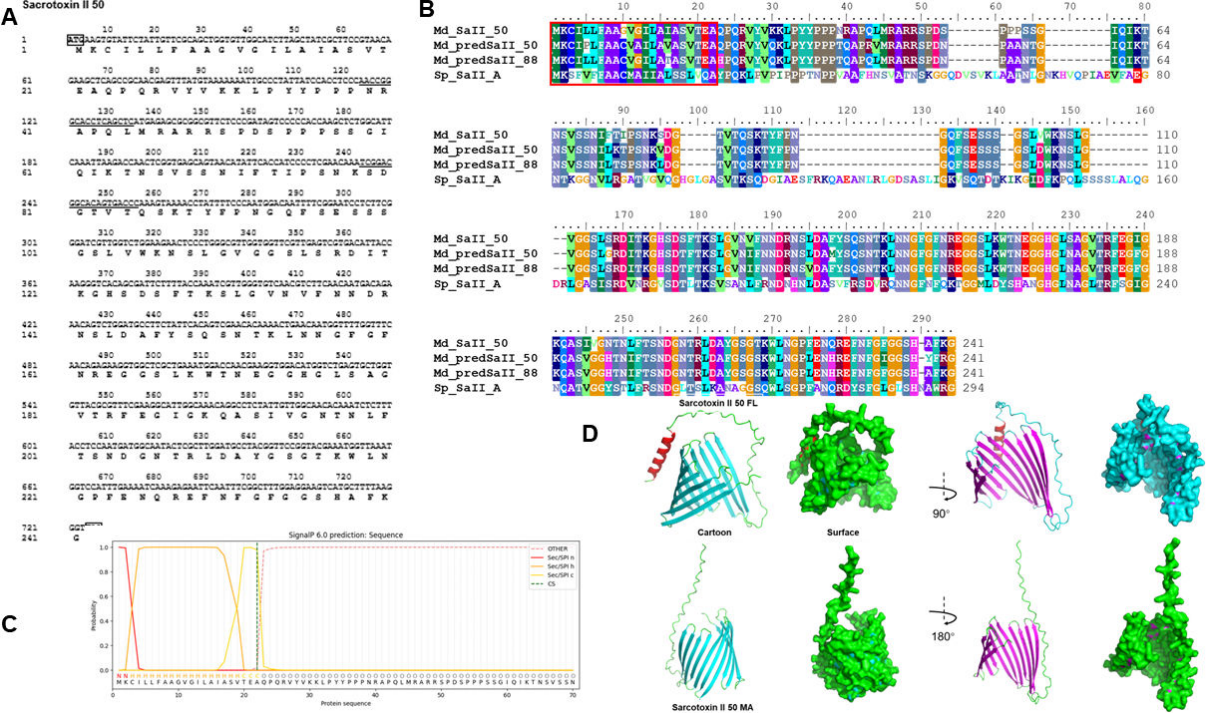
ORF: sequence alignment, and structural model of Sarcotoxin II 50. (**A**) The ORF of housefly Sarcotoxin II 50 was annotated by ORF Finder in DNAMAN. (**B**) Sequence alignment of *Musca domestica* Sarcotoxin II 50 with its homologs: XM_059132173.1 PREDICTED: *Musca domestica* sarcotoxin II-1-like (LOC131807150), NM_001308996.2 *Musca domestica* sarcotoxin II-1-like (LOC101893688), and M18873.1 Flesh fly (*Sarcophaga peregrina*) sarcotoxin II A. The red box represents the signal peptide predicted by SignalP 6.0 (**C**). Structural model of full-length Sarcotoxin II 50 (Sarcotoxin II 50 FL) with the signal peptide (UP) and without the signal peptide Sarcotoxin II 50 mature (Sarcotoxin II 50 MA) (DOWN), prepared using AlphaFold2 online software (**D**).

As an AMP, the insect Sarcotoxin was identified in flesh fly. Here, the housefly Sarcotoxin II 50 was isolated from bacteria-stimulated 3rd larvae cDNA pool. According to the template XM_059132173.1 PREDICTED: *Musca domestica* sarcotoxin II-1-like (LOC131807150) in NCBI, the full ORF was cloned and named Sarcotoxin II 50 (short for S50). However, there are some differences in the amino acid sequence of the ORF. The ORF codes for 241 amino acids (Fig. 1A), and the first 22 amino acids form its signal peptide (Fig. 1B and C). Sequence alignment showed that it is homologous with Sarcophaga peregrina Sarcotoxin II A (Fig. 1C). To further explore its characteristics, the structure of housefly Sarcotoxin II 50 was minimized using AlphaFold2 software. As shown in Fig. 1D, the full-length S50 with the signal peptide has an α-helix at the N-terminal. The C-terminal is more regular than N-terminal, with most of it consisting of β-sheets. It forms a semi-open barrel-shaped model. The mature form of S50 is without the signal peptide, which has lost the α-helix at the N-terminal. However, the barrel-plate model at the C-end is more compact, and it needs to be rotated 180 degrees to be fully displayed (Fig. 1D).

### **Effect of** Sarcotoxin II 50 transfection on cell viability

To assess whether transfection with Sarcotoxin II 50 (S50) affects host cell viability, CCK-8 assays were conducted at 24, 29, 36, and 48 h post-transfection. As shown in Fig. S2, there were no significant differences in cell viability between the S50-transfected cells and the empty vector control at any time point (*P*  > 0.05), indicating that S50 expression did not induce detectable cytotoxicity within the duration of the experiment.

### Sarcotoxin II 50 inhibits *K. pneumoniae* invasion of A549 and BEAS-2B cells

**Fig 2:**
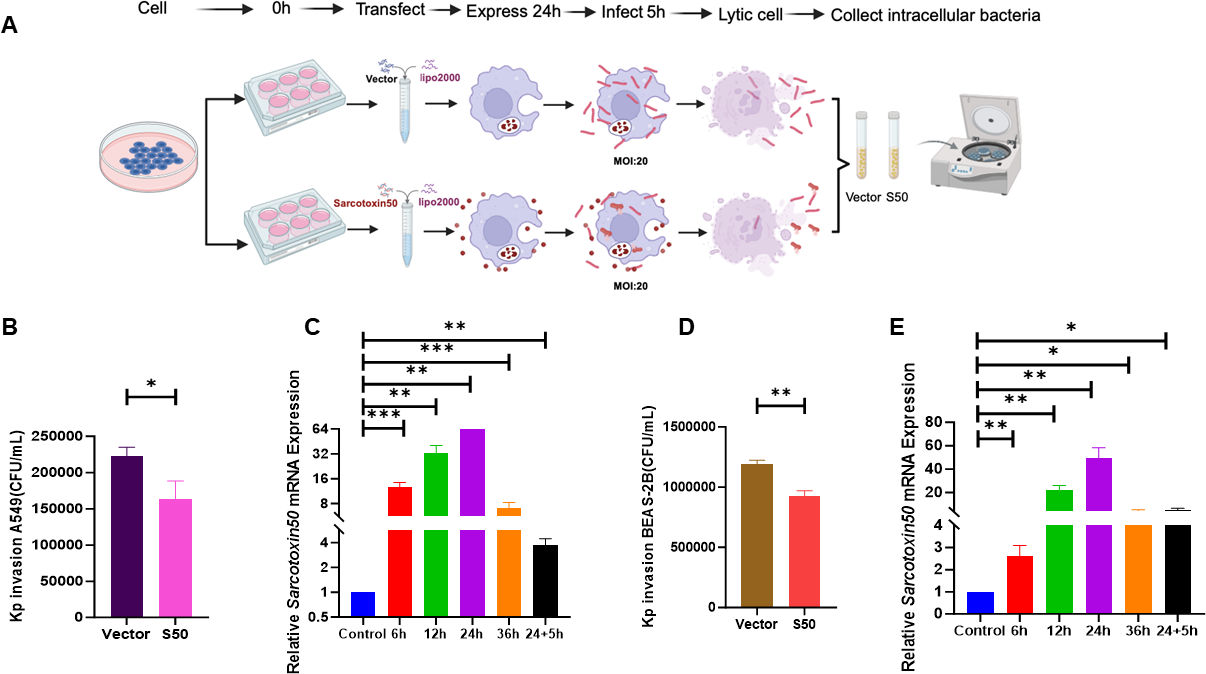
Sarcotoxin II 50 suppresses *K. pneumoniae* invasion of A549 and BEAS-2B cells. (**A**) Overview of the experimental workflow. (**B**) A549 and (**D**) BEAS-2B cells were transfected with Sarcotoxin II 50 for 24 h, followed by infection with *K. pneumoniae* at a multiplicity of infection (MOI) of 20 for 6 h. Intracellular bacterial loads were determined by serial dilution and colony-forming unit (CFU) counting. (**C**) A549 and (**E**) BEAS-2B cells were analyzed for Sarcotoxin II 50 mRNA expression at the indicated time points after transfection. Data are expressed as the mean ± standard deviation (SD) from three independent experiments (*n* = 3). Statistical significance was defined as *P < 0.05, **P < 0.01, and ***P < 0.001 compared with the control group.

To assess the antibacterial potential of Sarcotoxin II 50, a recombinant Sarcotoxin II 50–pcDNA3.1(+) expression vector was constructed and transfected into A549 and BEAS-2B cells using a eukaryotic heterologous expression system. Sarcotoxin II 50 mRNA levels were monitored at 1.5, 6, 12, 24, and 36 h post-transfection. Stable expression was observed in both cell lines, with peak transcript levels at 24 h (Fig. 2), confirming successful transfection and robust expression of Sarcotoxin II 50 in epithelial cells. Accordingly, 24 h post-transfection was selected as the optimal time point for subsequent antibacterial assays. Under these conditions, at a multiplicity of infection (MOI) of 20, Sarcotoxin II 50 markedly reduced the invasion of reference *K. pneumoniae* strains into both A549 and BEAS-2B cells. These findings collectively demonstrate that heterologous expression of Sarcotoxin II 50 in epithelial cells is effective and capable of inhibiting *K. pneumoniae* invasion.

### Validation of the antibacterial activity of Sarcotoxin II 50 against *K. pneumoniae*

**Fig 3:**
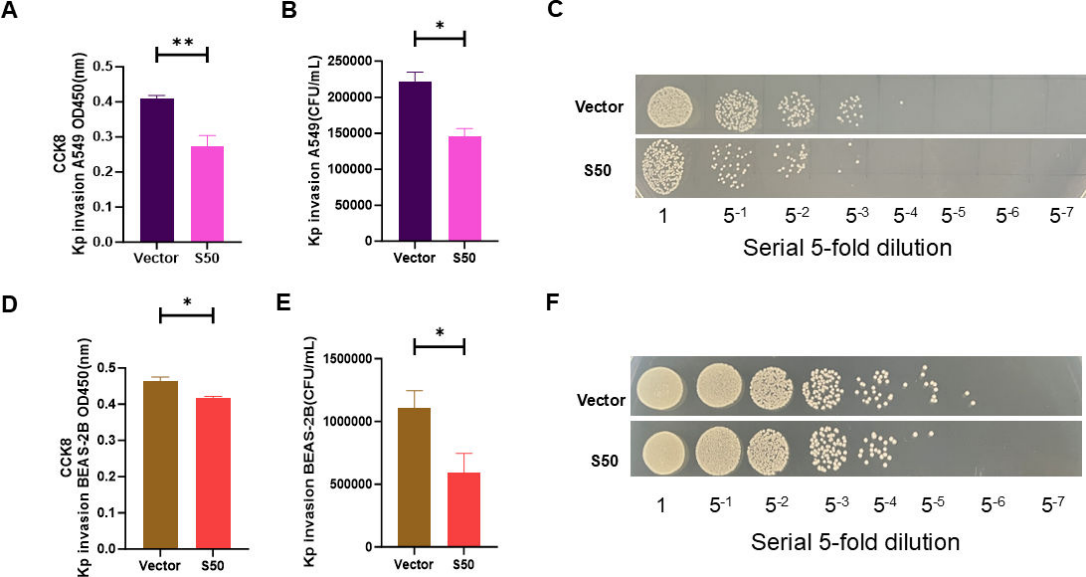
Confirmation of the antibacterial activity of Sarcotoxin II 50 against *K. pneumoniae*. A549 and BEAS-2B cells were transfected with Sarcotoxin II 50 for 24 h and then infected with *K. pneumoniae* at a multiplicity of infection (MOI) of 20. Host cell viability was evaluated using the CCK-8 assay in A549 (**A**) and BEAS-2B (**D**) cells. The intracellular bacterial loads in A549 (**B and C**) and BEAS-2B (**E and F**) cells were quantified by colony-forming unit (CFU) counting. Data are presented as the mean ± standard deviation (SD) from three independent experiments (*n* = 3). Statistical significance was defined as *P < 0.05, **P < 0.01, and ***P < 0.001 compared with the control group.

**Fig 4:**
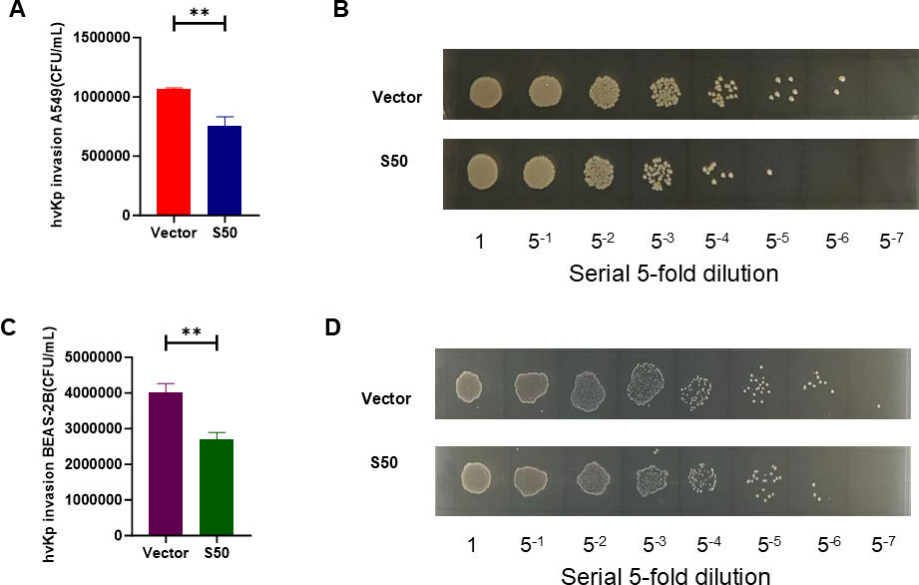
Validation of the antibacterial activity of Sarcotoxin II 50 against hvKP 26,020. A549 and BEAS-2B cells were transfected with Sarcotoxin II 50 for 24 h, followed by infection with hvKP 26,020 at a multiplicity of infection (MOI) of 20. Cell viability was assessed using the CCK-8 assay for A549 (**A**) and BEAS-2B (**C**) cells. Intracellular bacterial load was quantified by colony-forming unit (CFU) counting for A549 (**B**) and BEAS-2B (**D**) cells. Data represent mean ± SD (*n* = 3). Statistical significance: *P < 0.05, **P < 0.01 compared with the control group.

To comprehensively evaluate the antibacterial effect of Sarcotoxin II 50, both CCK-8 assays and colony-forming unit (CFU) counts were conducted. Sarcotoxin II 50 significantly inhibited the intracellular proliferation of standard *K. pneumoniae* in A549 cells (Fig. 3A) and BEAS-2B cells (Fig. 3D). Consistent with these findings, CFU enumeration confirmed a marked reduction in bacterial load in both A549 (Fig. 3B and E) and BEAS-2B cells (Fig. 3C and F). To further investigate the inhibition effect, a similar experiment was also conducted with high virulence *K. pneumoniae* (hvKP 26,020). Notably, CFU counts and spot plating confirmed that Sarcotoxin II 50 retains strong antibacterial activity against hvKP 26,020 (Fig. 4).

### Inhibitory effect of Sarcotoxin II 50 on *K. pneumoniae* growth

**Fig 5:**
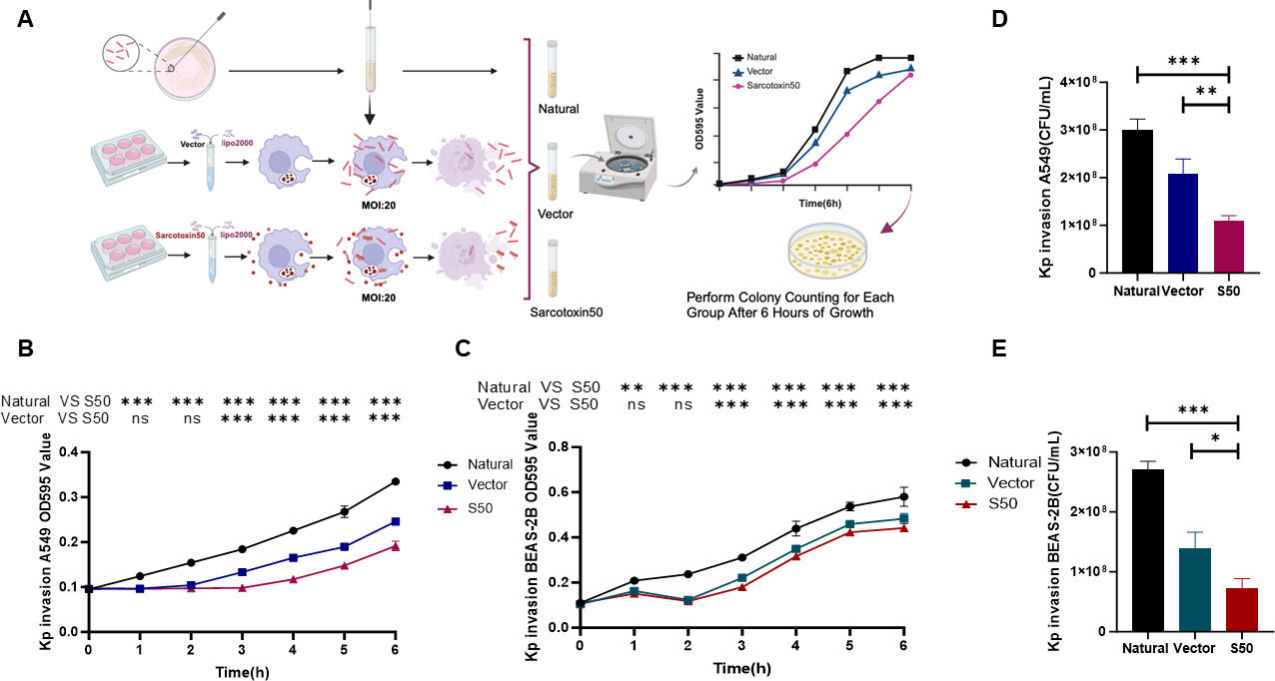
Sarcotoxin II 50 suppresses the intracellular growth of *K. pneumoniae*. (**A**) Schematic illustration of the experimental workflow. A549 (**B**) and BEAS-2B (**C**) cells were transfected with Sarcotoxin II 50 for 24 h and subsequently infected with *K. pneumoniae* at a multiplicity of infection (MOI) of 20. Intracellular bacteria were harvested 6 h after infection, and bacterial growth was monitored over the following 6 h. At the final time point, intracellular bacterial burdens were determined by colony-forming unit (CFU) enumeration (**D and E**). Data are presented as the mean ± standard deviation (SD) from three independent experiments (*n* = 3). Statistical significance was defined as *P < 0.05, **P < 0.01, and ***P < 0.001 compared with the control group.

Dynamic 6-hour growth curve analysis showed that Sarcotoxin II 50 effectively suppressed bacterial proliferation. In the A549 cell infection model, bacterial growth was substantially slowed in the Sarcotoxin II 50-treated group, with CFU counts at the 6-hour endpoint significantly decreased (Fig. 5B and D). Comparable results were observed in BEAS-2B cells, further confirming that Sarcotoxin II 50 inhibits *K. pneumoniae* growth (Fig. 5C and E).

### *In vivo* efficacy of Sarcotoxin II 50 against hypervirulent *K. pneumoniae* 26020

**Fig 6:**
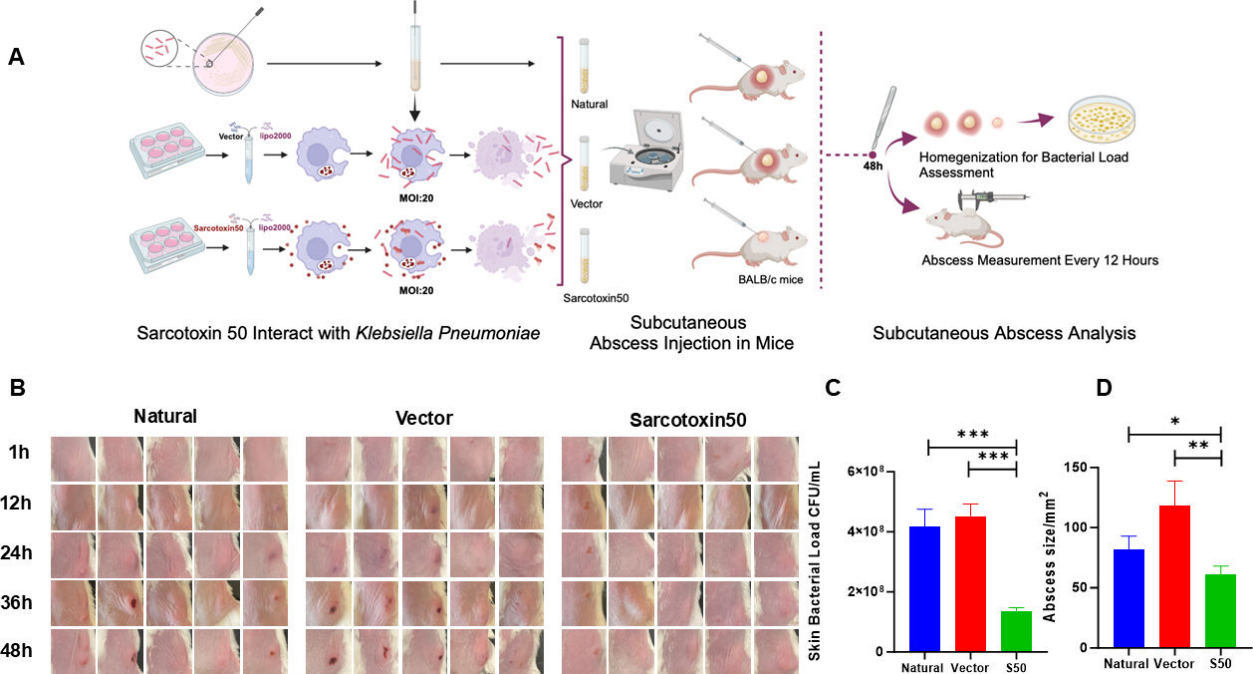
*In vivo* therapeutic efficacy of Sarcotoxin II 50 against hvKP 26,020. (**A**) Overview of the experimental workflow. A549 cells were transfected with Sarcotoxin II 50 for 24 h and then infected with hypervirulent *K. pneumoniae* 26,020 (hvKP 26,020) at a multiplicity of infection (MOI) of 20. Intracellular bacteria were subsequently collected from A549 cells and subcutaneously injected into mice. Abscess formation was evaluated 48 h post-injection (**B**), and both abscess area (**C**) and bacterial load in skin tissues (**D**) were quantified. Data are presented as the mean ± standard deviation (SD) from three independent experiments (*n* = 3). Statistical significance was defined as *P < 0.05, **P < 0.01, and ***P < 0.001 compared with the control group.

Due to the relatively low pathogenicity of the standard strain in the subcutaneous abscess mouse model, this study utilized the hypervirulent Kp clinical strain 26,020 to assess the *in vivo* efficacy of Sarcotoxin II 50. Within 48 h, abscess enlargement was substantially suppressed in the Sarcotoxin II 50-treated group (Fig. 6B). Both the abscess area and bacterial load, as measured by CFU counts, were significantly reduced (Fig. 6C and D), indicating that Sarcotoxin II 50 exerts strong *in vivo* antibacterial activity against hvKP 26,020.

### Anti-inflammatory activity of Sarcotoxin II 50 and modulation of capsular polysaccharide

**Fig 7:**
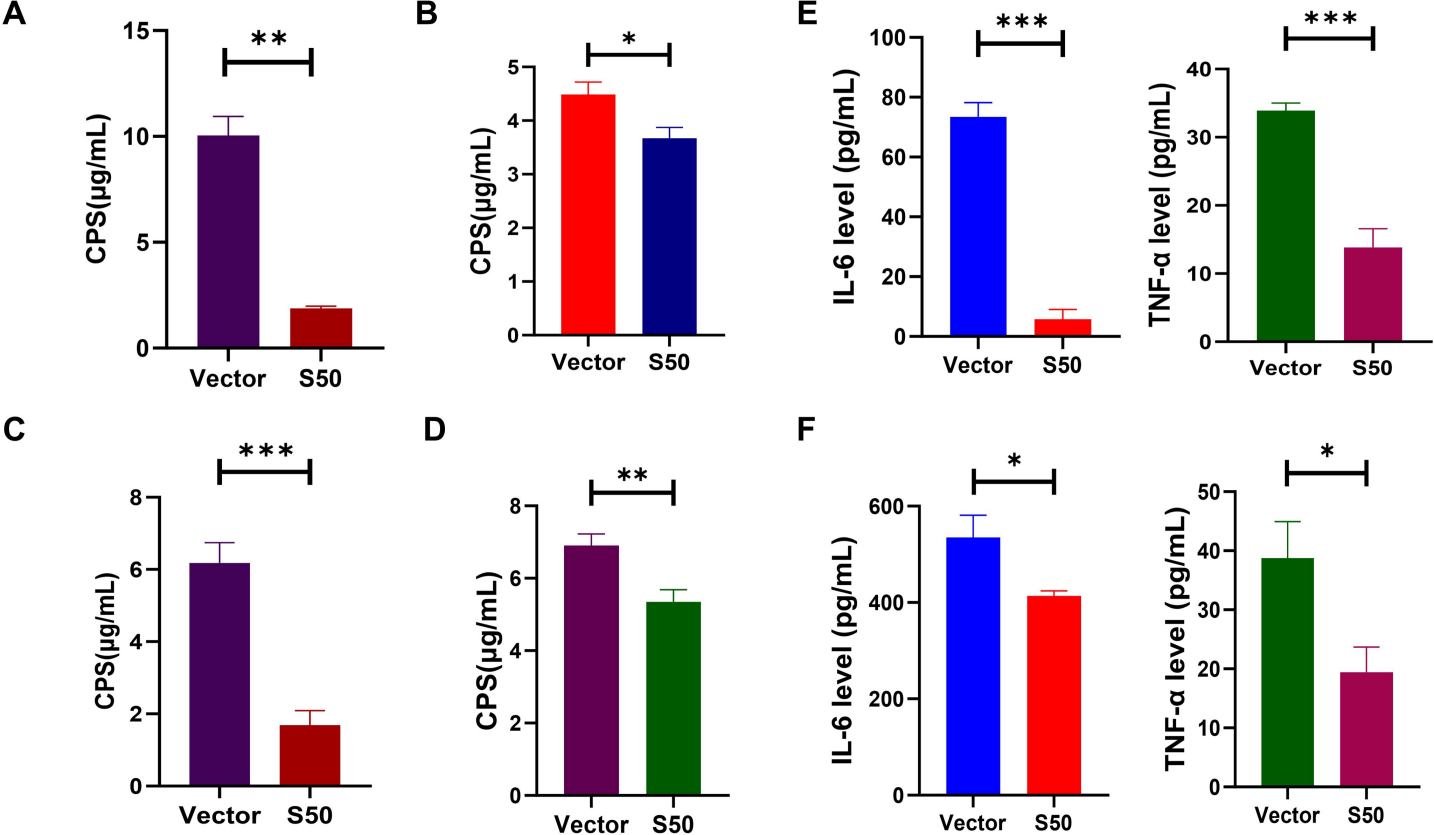
Sarcotoxin II 50 reduces inflammation and inhibits capsular polysaccharide production. A549 (**A**) and BEAS-2B (**C**) cells were transfected with Sarcotoxin II 50 for 24 h and then infected with *K. pneumoniae* at a multiplicity of infection (MOI) of 20. Six hours after infection, intracellular bacteria were harvested and normalized to the same cell number. Similar options as above and infect with hvKP 26,020 in A549 (**B**) and BEAS-2B (**D**). Capsular polysaccharide levels were quantified using the phenol–sulfuric acid assay. In Sarcotoxin II 50–transfected A549 (**E**) and BEAS-2B (**F**) cells, secretion of IL-6 and TNF-α was measured by ELISA. Data are presented as mean ± SD from three independent experiments (*n* = 3). Statistical significance: *P < 0.05, **P < 0.01, ***P < 0.001 compared with the control.

Further analysis showed that Sarcotoxin II 50 simultaneously dampens host inflammatory responses and reduces bacterial virulence. ELISA results revealed that, after infection, A549 cells transfected with Sarcotoxin II 50 produced significantly lower levels of the pro-inflammatory cytokines IL-6 and TNF-α (Fig. 7E). A comparable reduction was observed in BEAS-2B cells, supporting the conclusion that Sarcotoxin II 50 has clear anti-inflammatory activity (Fig. 7F). Moreover, intracellular levels of capsular polysaccharide (CPS) were markedly lower in the treated group than in the control group. Not only in normal strains (Fig. 7A and C), but also highly virulent strains (hvKP 26,020) (Fig. 7B and D) with obvious reduction of CPS upon S50 treatment in A549 and BEAS-2B. This reduction was consistent with decreased expression of the virulence-related genes *wzc* and *galF*, suggesting that Sarcotoxin II 50 weakens bacterial immune evasion by interfering with CPS biosynthesis (Fig. 7A through D).

### Sarcotoxin II 50 modifies the cellular morphology of *K. pneumoniae*

**Fig 8:**
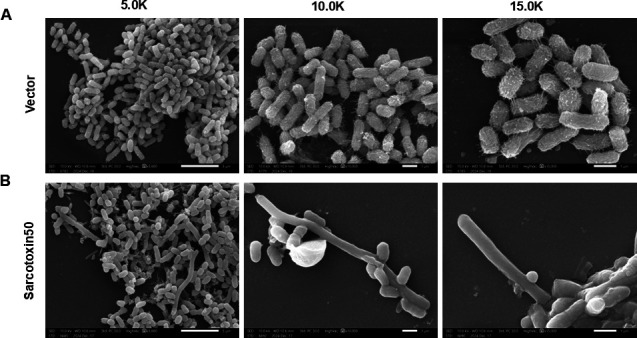
Scanning electron microscopy (SEM) analysis of A549 cells. A549 cells were transfected with Sarcotoxin II 50 for 24 h and then infected with *K. pneumoniae* at a multiplicity of infection (MOI) of 20. Following infection, cells were fixed with 2.5% glutaraldehyde, dehydrated through a graded ethanol series, dried, sputter-coated with gold, and observed by SEM. (**A**) Control group. (**B**) Sarcotoxin II 50–transfected group.

Scanning electron microscopy (SEM) revealed that Sarcotoxin II 50 treatment significantly altered the morphology of *K. pneumoniae*. Compared with the control group, bacterial density was reduced in the treated group, and some cells displayed surface protrusions, abnormal septum formation, and impaired cell division (Fig. 8A and B). Previous studies have shown that Sarcotoxin IIA can induce cell deformation and division arrest in Gram-negative bacteria by disrupting cell wall and septum synthesis (42, 50). Thus, the septal abnormalities observed here likely reflect a subset of cells experiencing division arrest or structural damage (51).

### Mechanistic insights into the effects of Sarcotoxin II 50 on *K. pneumoniae* pathogenicity and growth

**Fig 9:**
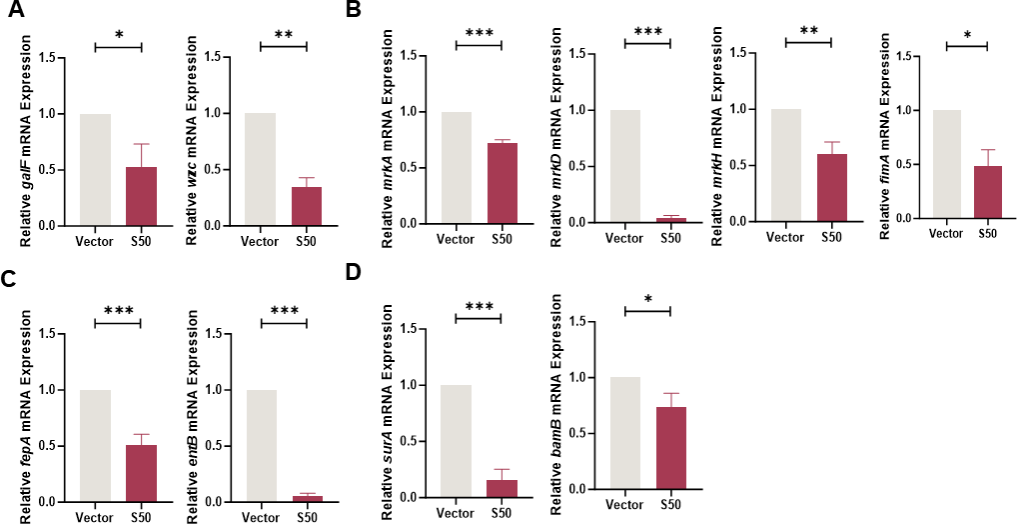
Effects of Sarcotoxin II 50 on the virulence of *K. pneumoniae*. A549 cells were transfected with Sarcotoxin II 50 for 24 h and then infected with *K. pneumoniae* at a multiplicity of infection (MOI) of 20 for 5 h. Intracellular bacteria were collected for RNA extraction, and the expression of virulence-associated genes was analyzed by quantitative real-time PCR (qRT-PCR), including capsule polysaccharide synthesis–related genes *wzc* and *galF* (**A**); fimbrial synthesis–related genes *mrkA*, *mrkD*, *mrkH*, and *fimA* (**B**); iron transport–related genes *fepA* and *entB* (**C**); and outer membrane protein biogenesis–related genes *surA* and *bamB* (**D**). The 16S rRNA gene served as an internal control. Data are presented as the mean ± standard deviation (SD) from three independent experiments (*n* = 3). Statistical significance was determined using an unpaired *t* test: *P < 0.05, **P < 0.01, ***P < 0.001 versus the vector control group.

To investigate the antibacterial mechanism of Sarcotoxin II 50, its effects on both *K. pneumoniae* pathogenicity and growth regulation were examined. At the virulence level, Sarcotoxin II 50 markedly suppressed key intracellular virulence factors in A549 cells. Expression of capsular polysaccharide (CPS) biosynthesis genes, including *galF* and *wzc*, was downregulated, impairing CPS assembly and thereby reducing the bacterium’s ability to evade host immune defenses (52) (Fig. 9A). Genes encoding type I pili (*mrkA*, *mrkD*, and *mrkH*) and type III pili (*fimA*) were also suppressed, resulting in diminished bacterial adhesion (Fig. 9B). Additionally, expression of iron transporter genes (*fepA* and *entB*) was decreased, limiting iron acquisition, inducing bacterial metabolic arrest, and consequently attenuating virulence (53, 54) (Fig. 9C). Outer membrane protein (OMP) synthesis genes (*surA* and *bamB*) were likewise downregulated, disrupting OMP-mediated mechanisms of host invasion (Fig. 9D) (55).

**Fig 10:**
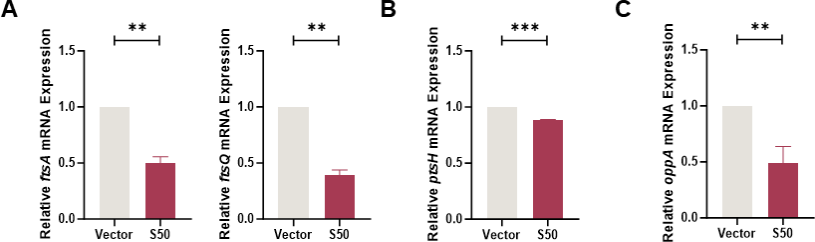
Effects of Sarcotoxin II 50 on cell division in *K. pneumoniae*. Quantitative real-time PCR (qRT-PCR) was used to assess the transcriptional levels of cell division–related genes *ftsA* and *ftsQ* (**A**), the phosphotransferase system gene *ptsH* (**B**), and the periplasmic oligopeptide transport regulatory gene *oppA* (**C**). The 16S rRNA gene served as an internal control. Data are presented as the mean ± standard deviation (SD) from three independent experiments (*n* = 3), and statistical analysis was performed using an unpaired *t*-test. Significance levels: *P < 0.05, **P < 0.01, ***P < 0.001 compared with the vector control group.

**Fig 11:**
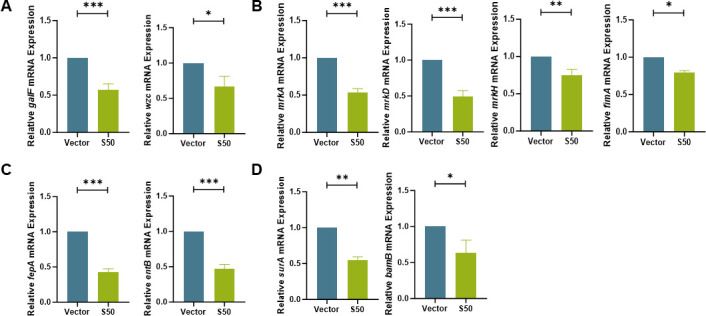
Effects of Sarcotoxin II 50 on the virulence of intracellular *K. pneumoniae* in BEAS-2B cells. BEAS-2B cells were transfected with Sarcotoxin II 50 for 24 h and then infected with *K. pneumoniae* at a multiplicity of infection (MOI) of 20 for 5 h. Intracellular bacteria were collected for RNA extraction, and the expression of virulence-associated genes was analyzed by quantitative real-time PCR (qRT-PCR), including capsule polysaccharide synthesis–related genes *galF* and *wzc* (**A**); fimbrial synthesis–related genes *mrkA*, *mrkD*, *mrkH*, and *fimA* (**B**); iron transport–related genes *fepA* and *entB* (**C**); and outer membrane protein biogenesis–related genes *surA* and *bamB* (**D**). The 16S rRNA gene served as an internal control. Data are expressed as the mean ± standard deviation (SD) from three independent experiments (*n* = 3). Statistical significance was determined using an unpaired *t* test: *P < 0.05, **P < 0.01, ***P < 0.001 versus the vector control group.

**Fig 12:**
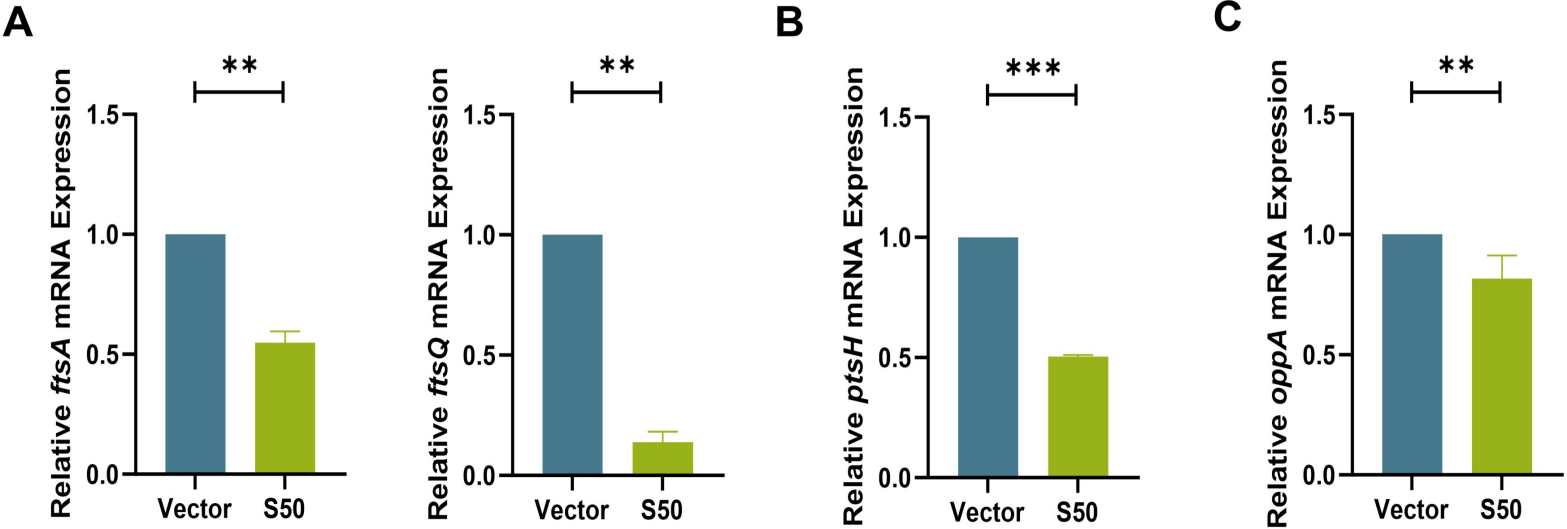
Effects of Sarcotoxin II 50 on cell division of intracellular *K. pneumoniae* in BEAS-2B cells. Quantitative real-time PCR (qRT-PCR) was used to assess the transcriptional levels of the cell division–related genes *ftsA* and *ftsQ* (**A**), the phosphotransferase system gene *ptsH* (**B**), and the periplasmic oligopeptide transport regulatory gene *oppA* (**C**). The 16S rRNA gene served as an internal control. Data are presented as the mean ± standard deviation (SD) from three independent experiments (*n* = 3), and statistical analysis was performed using an unpaired *t*-test. Significance levels: *P < 0.05, **P < 0.01, ***P < 0.001 versus the vector control group.

In regulating bacterial growth, Sarcotoxin II 50 markedly suppresses the proliferation of *Klebsiella pneumoniae* by acting on multiple targets in a coordinated manner. First, the expression of cell division genes *ftsA* and *ftsQ* was markedly downregulated, resulting in abnormal septum formation and impaired cell division (40) (Fig. 10A). Second, Sarcotoxin II 50 significantly reduced the expression of *ptsH* (Fig. 10B). The *ptsH* gene encodes HPr, a core component of the phosphoenolpyruvate-dependent phosphotransferase system (PTS), which serves as the primary pathway for sugar uptake and phosphorylation in bacteria. HPr functions as a key phosphate carrier, transferring phosphate groups from enzyme I (EI) to the sugar-specific enzyme II complex, thereby facilitating transport and phosphorylation of substrates, such as hexoses and disaccharides. Through this process, *ptsH* plays a central role in maintaining carbon source utilization, energy metabolic homeostasis, and overall metabolic adaptability in bacteria. Beyond its metabolic functions, *ptsH* has been increasingly linked to stress regulation and persister cell formation in *K. pneumoniae*. Transcriptomic analyses indicate that *ptsH* expression is significantly upregulated in persister cells under antibiotic stress and declines during resuscitation. Further studies show that *ptsH* deletion mutants exhibit markedly reduced persister formation under antibiotic pressure, suggesting that HPr may promote persister generation by modulating metabolic status and stress responses. Potential mechanisms include reduced intracellular cyclic AMP (cAMP) levels, decreased reactive oxygen species (ROS) accumulation, and enhanced antioxidant capacity, collectively facilitating entry into a low-metabolic, quasi-dormant state that increases antibiotic tolerance (56). In this study, the observed downregulation of *ptsH* by Sarcotoxin II 50 suggests that the peptide may disrupt bacterial energy metabolism and stress response networks, thereby inhibiting persister cell formation and increasing *K. pneumoniae* susceptibility to host defenses and antibiotic treatment. Additionally, Sarcotoxin II 50 significantly suppressed *oppA* expression (Fig. 10C). The *oppA* gene encodes the substrate-binding protein of the oligopeptide transport system, and its reduced expression may further compromise nutrient uptake, bacterial adhesion, and colonization. Importantly, in the BEAS-2B infection model, these gene expression changes closely mirrored the *in vitro* findings (Fig. 11 and 12), confirming that Sarcotoxin II 50 simultaneously interferes with cell division, metabolic regulation, and nutrient acquisition. Together, these multi-target effects synergistically inhibit *K. pneumoniae* colonization, persister formation, and proliferation.

## DISCUSSION

*K. pneumoniae* is a leading cause of hospital-acquired pneumonia (5, 57), and its hypervirulent strains (hvKP) represent a serious global public health threat due to their high pathogenicity and multidrug resistance (58). As conventional antibiotics lose efficacy, insect-derived antimicrobial peptides (AMPs) have attracted increasing attention for their broad-spectrum activity, high potency, and low risk of inducing resistance (59–62). The common housefly (*Sarcophaga peregrina*) possesses a sophisticated innate immune system capable of secreting antimicrobial peptides to combat invading pathogens. In this study, we identified Sarcotoxin II 50, a member of the Sarcotoxin IIA family, as being induced by body-wall injury in houseflies and systematically evaluated its inhibitory activity and mechanism against *K. pneumoniae*. Our results demonstrated that Sarcotoxin II 50 effectively suppressed the growth of both the standard strain (27,736) and the hypervirulent strain (26,020) *in vitro* and *in vivo*. Scanning electron microscopy revealed disruption of bacterial septa and reduction of surface pili, consistent with previous observations of morphological damage in *Escherichia coli* induced by Sarcotoxin IIA (42). Beyond growth inhibition, Sarcotoxin II 50 downregulated key genes involved in capsular polysaccharide biosynthesis (*wzc*, *galF*) and pili formation (*fimA*, *mrkA*, *mrkD*, *mrkH*), collectively impairing *K. pneumoniae* adhesion and colonization. Moreover, in transfected host cells, bacterial infection triggered reduced expression of inflammatory cytokines, reflecting the peptide’s ability to attenuate bacterial pathogenicity. Overall, this study enhances understanding of the antimicrobial mechanisms of the Sarcotoxin IIA family and provides a theoretical foundation, as well as potential translational prospects, for developing natural peptide–based anti-infective agents that combine antibacterial and anti-virulence properties.

In this study, Sarcotoxin II 50 was heterologously expressed in a eukaryotic system to facilitate appropriate post-translational modifications, particularly glycosylation, which are often absent in prokaryotic expression systems and may compromise protein stability and biological activity. This strategy, therefore, helps preserve the functional integrity of Sarcotoxin II 50. Moreover, heterologous expression reduces the likelihood of activating Sarcotoxin II 50–associated signaling pathways, thereby minimizing potential experimental interference (63). Importantly, intracellular expression of Sarcotoxin II 50 showed good biosafety, as no significant effects on host cell viability were observed within 24–48 h after transfection, indicating a lack of obvious cytotoxicity and supporting the validity of subsequent infection experiments (Fig. S2). Expression kinetic analysis showed that Sarcotoxin II 50 reached maximal expression at 24 h post-transfection and remained at relatively high intracellular levels even 5 h after *Klebsiella pneumoniae* infection (Fig. 2). This sustained intracellular presence may underlie its prolonged antibacterial activity and contrasts with most synthetic antimicrobial peptides, which typically have short half-lives and are rapidly degraded in mammalian systems (64). Functional studies further demonstrated that eukaryotically expressed Sarcotoxin II 50 significantly impaired the ability of *K. pneumoniae* to invade epithelial cells and markedly reduced both intracellular bacterial load and viability, indicating that Sarcotoxin II 50 not only interferes with bacterial invasion but also exerts potent intracellular antibacterial activity (Fig. 3 and 4). Consistent with previous reports on insect-derived antimicrobial peptides, such as defensins and cecropins, which inhibit bacterial adhesion and invasion and modulate host–pathogen interactions (32, 65, 66), these findings further support the notion that insect antimicrobial peptides exert multifaceted protective functions during bacterial infection (Fig. 3).

In the 6-hour growth curve assay, Sarcotoxin II 50 was found to markedly suppress the proliferation of intracellular *K. pneumoniae*. In both A549 and BEAS-2B cells, bacterial growth was substantially restricted in the treatment groups, and CFU counts at the final time point were significantly lower than those in the control groups (Fig. 5). These results are consistent with previous reports showing that antimicrobial peptides can exert bacteriostatic effects by disrupting key processes involved in bacterial growth (67).

The ability of Sarcotoxin II 50 to attenuate *K. pneumoniae* pathogenicity was evaluated using a murine subcutaneous infection model. In this experiment, intracellular bacteria isolated from A549 cells were used for subcutaneous inoculation. Because standard strains exhibit relatively low virulence *in vivo*, the hypervirulent strain hvKP 26,020 was selected to more rigorously evaluate the antibacterial efficacy of Sarcotoxin II 50. This strain, characterized by its robust capsular polysaccharide production and virulence gene repertoire, reliably induces pronounced subcutaneous abscesses. Within 48 h after infection, treatment with Sarcotoxin II 50 significantly reduced abscess formation, accompanied by significant decreases in both bacterial burden and abscess size (Fig. 6).

To clarify how Sarcotoxin II 50 inhibits *K. pneumoniae*, we systematically examined its effects on intracellular capsular polysaccharide (CPS) production and host inflammatory responses. CPS is known to play a central role in immune evasion, resistance to phagocytosis, and the development of antimicrobial tolerance in *K. pneumoniae* (52), and is therefore considered a key determinant of bacterial virulence (68). The marked reduction in CPS levels observed after Sarcotoxin II 50 treatment indicates that the peptide likely weakens bacterial survival and persistence within host cells by interfering with capsular polysaccharide synthesis, thereby reducing pathogenic potential (Fig. 7A through D). Because *K. pneumoniae* infection can strongly stimulate host immune responses and provoke inflammation (47, 69), we also evaluated the production of pro-inflammatory cytokines. Under infection conditions, cells transfected with Sarcotoxin II 50 showed significantly lower levels of IL-6 and TNF-α compared with controls (Fig. 7E and F). Although inflammation is an essential component of host defense against bacterial invasion (70), excessive or prolonged inflammatory responses can cause tissue damage and exacerbate disease. These findings, therefore, suggest that Sarcotoxin II 50 not only suppresses bacterial virulence but also helps to moderate infection-associated inflammatory reactions. To further support these mechanistic insights, scanning electron microscopy (SEM) was used to visualize bacterial morphological changes induced by Sarcotoxin II 50. SEM images showed that treated *K. pneumoniae* cells displayed fewer surface fimbriae, smoother outer membranes, and, in some cases, structural features consistent with impaired cell division (Fig. 8). These morphological changes closely align with the observed inhibition of CPS synthesis and disruption of bacterial growth, providing direct structural evidence that Sarcotoxin II 50 attenuates *K. pneumoniae* pathogenicity by targeting capsular polysaccharide production and growth regulation.

**Fig 13:**
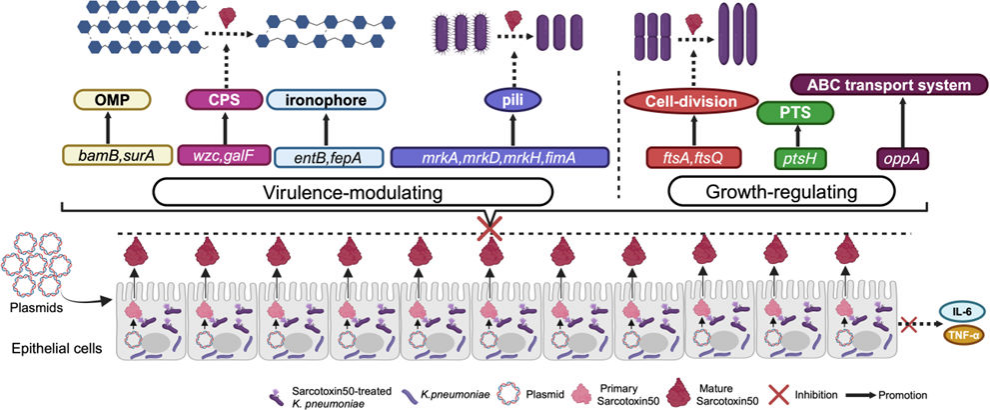
Proposed mechanism of Sarcotoxin II 50–mediated inhibition of *K. pneumoniae* invasion into lung epithelial cells. Sarcotoxin II 50 effectively inhibits *K. pneumoniae* invasion of lung epithelial cells through a three-pronged synergistic mechanism. In terms of virulence regulation, it suppresses capsule synthesis by downregulating the key virulence genes *wzc* and *galF*, thereby blocking capsule polysaccharide production. This enhances complement deposition on the bacterial surface and increases bacterial susceptibility. For adhesion regulation, Sarcotoxin II 50 reduces the expression of critical fimbrial regulatory genes *mrkA*, *mrkD*, *mrkH*, and *fimA*, impairing bacterial adhesion and biofilm formation—both essential for colonization and persistent survival. Regarding bacterial growth, Sarcotoxin II 50 disrupts cell division by interfering with septum formation, causing abnormal division and ultimately inhibiting bacterial proliferation.

To better understand how Sarcotoxin II 50 inhibits *K. pneumoniae*, we combined our experimental findings with evidence from previous studies to examine its regulatory effects on genes associated with bacterial virulence and growth. Our results showed that Sarcotoxin II 50 significantly reduced the expression of a broad set of virulence-related genes in intracellular *K. pneumoniae* within A549 cells, including genes involved in capsular polysaccharide (CPS) synthesis, fimbrial assembly, siderophore production, and outer membrane protein formation. These observations are in line with earlier reports demonstrating the central roles of these factors in bacterial pathogenicity. Capsular polysaccharides are known to promote immune evasion and contribute to severe lung infections in animal models (71, 72), while also enhancing antibiotic resistance by stabilizing biofilm structures (73, 74). Among the CPS-associated genes, *galF* and *wzc* are key regulators of capsule biosynthesis and are involved in immune evasion, adhesion, and cell division (75). Their downregulation by Sarcotoxin II 50 suggests that the peptide may disrupt capsule formation and capsule–biofilm interactions, thereby limiting bacterial colonization (Fig. 9A). Fimbrial genes, including *mrkA*, *mrkD*, *mrkH*, and *fimA*, are also essential for bacterial attachment and colonization (Fig. 9B). Type III fimbriae are mainly composed of the major pilin encoded by *mrkA* and the adhesin encoded by *mrkD*, which together promote biofilm formation and host cell adherence (76, 77). The transcriptional regulator *mrkH*, which responds to c-di-GMP, controls expression of the *mrk* operon (78), while *fimA*, the principal structural gene of type I fimbriae, has recently been implicated in *K. pneumoniae* colonization and proposed as a potential vaccine target (79). The simultaneous suppression of these fimbrial genes by Sarcotoxin II 50 therefore indicates a reduced capacity for fimbriae-mediated adhesion and early colonization, weakening the bacterium’s ability to stably attach to host cell surfaces. In addition, the decreased expression of the siderophore-related genes *entB* and *fepA* is consistent with previous findings showing that impaired iron acquisition diminishes bacterial virulence (Fig. 9C). Similarly, downregulation of the outer membrane protein–associated genes *surA* and *bamB* may compromise outer membrane integrity and interfere with the proper assembly of virulence-associated proteins (53, 54) (Fig. 9D). Notably, Sarcotoxin II 50 also reduced the expression of the cell division genes *ftsQ* and *ftsA*, as well as the oligopeptide transport gene *oppA*, indicating that the peptide affects not only virulence factor production but also bacterial growth and nutrient uptake (Fig. 10). An equivalent inhibitory effect of S50 was likewise observed in BEAS-2B cells infected with *K. pneumoniae* (Fig. 11 and 12). Taken together, these results suggest that Sarcotoxin II 50 suppresses *K. pneumoniae* through multiple coordinated mechanisms, simultaneously targeting capsule synthesis, fimbrial adhesion, and growth- and division-related pathways. This multifaceted mode of action provides a strong mechanistic basis for the future development of antimicrobial peptide–based therapeutic strategies (Fig. 13).

Based on our findings, Sarcotoxin II 50 appears to display stronger inhibitory activity against *K. pneumoniae* than other members of the Sarcotoxin IIA family, effectively targeting both hypervirulent and standard strains. The data suggest that Sarcotoxin II 50 reduces the invasive capacity of *K. pneumoniae* by limiting capsular polysaccharide production and downregulating key determinants required for bacterial growth.

## Data Availability

The data of this study are available in the paper and its supplemental materials.

## SUPPLEMENTAL MATERIAL

Supplemental material spectrum.00962-25-s0001.docx Tables S1 and S2; Fig. S1 and S2.

ASM does not own the copyrights to Supplemental Material that may be linked to, or accessed through, an article. The authors have granted ASM a non-exclusive, world-wide license to publish the Supplemental Material files. Please contact the corresponding author directly for reuse.
