# Supplementary material for: Mechanistic insights into the inhibitory effects of eukaryotically expressed Sarcotoxin II 50 on Klebsiella pneumoniae invasion into pulmonary epithelial cells
Source: Microbiol Spectr. 2026 Mar 17;14(4):e00962-25. doi: 10.1128/spectrum.00962-25 (PMC13055303; doi:10.1128/spectrum.00962-25)
Supplement: Supplemental material — Tables S1 and S2; Fig. S1 and S2. [file spectrum.00962-25-s0001.docx]

**Supplementary Table 1** Primer list of Sarcotoxin90 used here.

| Gene | Sequence (5' to 3') | Length  (bp) | Usage |
| --- | --- | --- | --- |
| Sarcotoxin90 F | TCGTTCTCCCGATTGTCACTC | 21 | Real time-PCR |
| Sarcotoxin90 R | GTTCCGATCATTGTTGAAGATGT | 23 |  |
| *gapdh* F | GACCTGACCTGCCGTCTA | 18 |  |
| *gapdh* R | AGGAGTGGGTGTCGCTGT | 18 |  |

**Supplementary Table 2** Realtime PCR primer list of Klebsiella pneumoniae virulence related genes.

| Gene | Sequence (5' to 3') | Length  (bp) | Usage |
| --- | --- | --- | --- |
| *galF* F | CGGTCGCAGGACTAGGCAT | 19 | Real time -PCR |
| *galF* R | GCTTCCAGCTCGTAGGAGGTG | 21 |  |
| *wzc* F | GATGCAGATATGCGACGAGG | 20 |  |
| *wzc* R | TCCGCTGGATTTGGTGGT | 18 |  |
| *mrkA* F | GAACTGGACCGGCGGTAA | 18 |  |
| *mrkA* R | TCACCCGGGATGATTTTGTT | 20 |  |
| *mrkD* F | GTCTTTTCGTCCCGGGTATATAAC | 24 |  |
| *mrkD* R | CCACATCGACATTCATATTTTTCC | 24 |  |
| *mrkH* F | TCAGACCCATCGCAAAATCC | 20 |  |
| *mrkH* R | CGTAAACGAAAGCGGGGAT | 19 |  |
| *fimA* F | CGTCGGTTTCAACATCCAG | 19 |  |
| *fimA* R | GGTGGTATTGCTGCTGTCG | 20 |  |
| *fepA* F | CAGCGGATGAAGGATAATGCC | 21 |  |
| *fepA* R | GGACAGGTTAAGGGACGGACTC | 22 |  |
| *entB* F | ATATCGGCTGCATGACCACC | 20 |  |
| *entB* R | GCACCGAATCCAGACCGTAGT | 21 |  |
| *ftsQ* F | CAGGTGCTGGCAAAGGATAA | 20 |  |
| *ftsQ* R | TGCTGAAGAACCGGGTAGAGT | 21 |  |
| *ftsA* F | TCTGCGTGGTGGATATTGG | 19 |  |
| *ftsA* R | AACCGTGACGAACCTTGATG | 20 |  |
| *oppA* F | ACCGCCCAGGACTTCGTTT | 19 |  |
| *oppA* R | TGGCTCGCTGAGGGTGACTT | 20 |  |
| *ptsH* F | CCCTGCTGCTCAGTTTGTT | 19 |  |
| *ptsH* R | TTGCCGTTGGAGGTTACAG | 19 |  |
| *surA* F | AAAGCCATCGCTGATATTGC | 19 |  |
| *surA* R | AGCTTCATCAGTGCATCACG | 21 |  |
| *bamB* F | GCCTGCACCAGCAACTTATC | 20 |  |
| *bamB* R | AAGGTCTGGTGAAAGCGATG | 20 |  |
| *16S* F | AGAGCAAGCGGACCTCATAAA | 21 |  |
| *16S* R | AACGTATTCACCGTGACATTCTG | 23 |  |

**D**

**C**

**B**

**A**

**Supplementary Figure 1** Effect of eukaryotically expressed Sarcotoxin II 50 (S50) on the intracellular invasion of hvKP26020 in A549 and BEAS-2B cells. To preliminarily assess the dose-dependent therapeutic potential of S50, cells were transfected with S50 plasmid at plasmid-to-transfection reagent ratios of 1:1 and 1:3, followed by infection with hvKP26020. No inhibitory effect on bacterial invasion was observed at either ratio. Notably, the commonly used ratio of 1:2 (plasmid: transfection reagent) showed better antibacterial effect in experiments. (A, B) Transfection at 1:1 ratio: (A) A549 cells, (B) BEAS-2B cells. (C, D) Transfection at 1:3 ratio: (C) A549 cells, (D) BEAS-2B cells. Data represent mean ± SD (n = 3). Statistical significance: *P < 0.05, compared with the control group.

**B**

**A**

**Supplementary Figure 2**. Effects of Sarcotoxin II 50 transfection on host cell viability. (A) Viability of A549 cells at 24, 29, 36, and 48 hours post-transfection with S50. (B) Viability of BEAS-2B cells at 24, 29, 36, and 48 hours post-transfection with S50. Cell viability was measured using the CCK-8 assay. No significant differences were observed between the S50-transfected groups and the empty vector control at any time point (P > 0.05), indicating that expression of S50 does not cause appreciable cytotoxicity within the tested period. Data are presented as mean ± SD of three independent experiments (n = 3). Significance levels: *P < 0.05, **P < 0.01, ***P < 0.001 compared with the control group.
